# The evolution and application of the medical evidence chain

**DOI:** 10.3389/fmed.2025.1690197

**Published:** 2025-12-11

**Authors:** Yanan Bai, Wanying Wang, Jiajia Duan, Xue Wang, Mingjing Jiang, Han Ni, Lu Sun, Chuanxin Liu, Ding Li

**Affiliations:** 1 School of Law, Xiamen University, Xiamen, China; 2 Luoyang Key Laboratory of Clinical Multiomics and Translational Medicine, Henan Key Laboratory of Rare Diseases, Endocrinology and Metabolism Center, The First Affiliated Hospital, and College of Clinical Medicine of Henan University of Science and Technology, Luoyang, China; 3 Department of Clinical Laboratory, The First Affiliated Hospital, College of Clinical Medicine of Henan University of Science and Technology, Luoyang, China; 4 State Key Laboratory of Component-based Chinese Medicine, Tianjin University of Traditional Chinese Medicine, Tianjin, China; 5 School of Law, Zhongyuan University of Technology, Zhengzhou, China; 6 College of Chinese Materia Medica and Food Engineering, Shanxi University of Chinese Medicine, Jinzhong, China; 7 Department of Pharmacy, The Affiliated Cancer Hospital of Zhengzhou University and Henan Cancer Hospital, HNHC Key Laboratory of Anti-tumor Drug Research, Henan Engineering Research Center for Tumor Precision Medicine and Comprehensive Evaluation, Zhengzhou, China

**Keywords:** evidence chain, connecting points, corroboration, evidence-based medicine, GRADE, integrated evidence chain

## Abstract

**Introduction:**

Since the advent of evidence-based medicine, the decision-making process in disease diagnosis and treatment has exhibited fundamental principles that align closely with the core tenets of evidence chain theory in the legal field. Based on this, this paper aims to explore the underlying logic and practical value of transferring evidence chain theory from law to medicine, focusing on its significance in enhancing the scientific rigor and standardization of clinical decision-making.

**Methods:**

Through systematic retrieval of existing literature in the fields of jurisprudence and medicine, this paper reviewed the theoretical evolution and practical applications of the evidence chain in jurisprudence, outlined the grading criteria for medical evidence, and elaborated on the construction methods and application examples of the integrated evidence chain and toxicological evidence chain.

**Results:**

The mutual corroboration among evidence materials significantly enhances the reliability and validity of conclusions. The theory of evidence chains plays a pivotal role in evaluating drug efficacy, elucidating toxicological mechanisms, optimizing clinical pathways, and designing high-quality clinical trials.

**Conclusion:**

The interdisciplinary application of the evidence chain theory contributes to advancing the standardization and normalization of evidence-based medical practice, thereby enhancing the quality of clinical decision-making and public health management.

## 1 Introduction

The evidence chain is a concept centered on the systematic use of evidence. It refers to a comprehensive structure formed through the corroboration, reinforcement, or aggregation of multiple pieces of evidence to substantiate a particular fact. Evidence plays a critical role in everyday reasoning, and the factual claims it supports are generally regarded as highly authentic and credible. Scholarly discussions on evidence and evidence chains originate primarily in the domain of evidence law within jurisprudence. In the context of criminal, civil, and administrative litigation, the concept of evidence is also largely shaped by legal definitions. Similarly, the medical field requires foundational cognitive frameworks to examine issues in pathology, pharmacology, and toxicology (1). In this regard, the theoretical and practical advancements in jurisprudential studies of the evidence chain offer valuable insights for its application in medical research and practice (2). At its core, the evidence chain provides a methodological framework for understanding phenomena and verifying facts. In medical research, evidence obtained from *in vitro* experiments, clinical trials, and other scientific methods can be systematically integrated through an evidence chain to evaluate drug efficacy and safety. The construction of such chains plays a decisive role in the scientific interpretation and application of evidence materials for identifying medical facts. Accordingly, the development of evidence chain methodology in medicine remains closely linked to its jurisprudential foundations. This paper adopts an interdisciplinary perspective to trace the evolution and application of the evidence chain within the medical domain.

## 2 Evolution and application of the evidence chain theory in jurisprudence

Originally emerging in jurisprudence, the evidence chain was conceived as a practical tool for integrating and correlating diverse pieces of evidence to establish the factual truth in legal cases. It serves as a critical method for evidence-based determination (3). The evidence chain is constructed through the interrelations among pieces of case evidence, which often display distinct structural configurations.

### 2.1 Concept and characteristics of the evidence chain

In legal theory, the fundamental attributes of evidence are legality, relevance, and authenticity. Among these, relevance denotes the logical connection between the evidence content and the facts of the case. Within a consistent factual context, evidence often displays mutually reinforcing and corroborative relationships—that is, “connecting points” (4). The theory of relevance underpinning the evidence chain traces back to the former Soviet Union, where it was initially employed to assess the validity and applicability of circumstantial evidence in litigation (5). Early conceptualizations defined the evidence chain as a composite structure consisting of two or more distinct pieces of evidence, connected through “chain heads” and “chain bodies” that form objective linkages. These interconnections serve to mutually corroborate content, enhance overall credibility, and ultimately substantiate case facts (6). In judicial practice, the criterion of sufficient evidence is often represented through the interlocking configuration of an evidence chain, which reflects the factual circumstances of a case. During the process of reconstructing case facts, evidence chains are also characterized by attributes such as hierarchy and completeness (7). As jurisprudential inquiry into the evidence chain deepens, the conceptual elements of “chains” and “connecting points” have become focal points of theoretical analysis. Evaluative standards for the credibility of an evidence chain now emphasize the number and hierarchical organization of these connecting points (8). On this basis, evidence chains synthesize diverse types of evidence to construct a coherent and comprehensive representation of case facts (9).

Therefore, it is necessary to integrate the corroboration theory, which emphasizes mutual cross-referencing among pieces of evidence, with the evidence chain framework, in order to articulate the substantive corroborative relationships at the “connecting points” between evidentiary elements. This foundational understanding remains consistent even within the domain of evidence-based medicine (EBM) (10, 11).

### 2.2 Proposition and application of the corroboration theory of evidence

A key challenge lies in establishing appropriate criteria and methods for evaluating evidence—an unavoidable concern in the adoption and verification of factual content. Historically, although jurisdictions differ in their rules for determining evidence admissibility, all recognize the role of the adjudicator’s free evaluation of evidence in the fact-finding process. However, such free evaluation is often vulnerable to subjective bias, resulting in significant arbitrariness (12). To counter this, some scholars pioneered the corroborative evidence theory: It suggests that corroboration refers to the mutual verification and alignment among pieces of evidence (13), and it typically manifests as a coordinated relationship between different evidentiary materials (14), encompassing point-based corroboration and chain-based corroboration.

**Figure 1:**
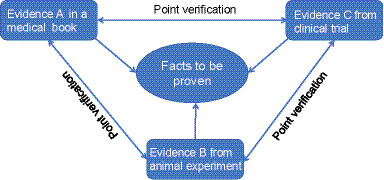
Diagram of point verification and evidence chain.

Therein, the meaning of “point-based corroboration” is the commonalities between single evidence and the other one. Sometimes, it can reflect from the commonalites or can be inferred to the same conclusion. In contrast, chain-based corroboration relies on the interconnection of intermediate facts, linking different evidence sets across chains to form a coherent system for substantiating the ultimate factual claims (15). The degree of mutual corroboration between pieces of evidence determines the number of connecting points; multiple points may exist between any two pieces of evidence. When evidence chains intersect and form a closed network, a standardized pattern can be identified across the case context (16). This theory promotes a holistic evaluation approach, focusing on the overall evidentiary profile rather than isolated fragments. Within the medical community, this integrated perspective—rooted in EBM—has gradually gained consensus, as illustrated in Figure 1.

In the medical context, demonstrating the therapeutic efficacy of a drug often begins with classical medical literature or authoritative texts. When experimental results from cellular or animal studies confirm effects recorded in these texts, a point-based corroboration is formed. Such interlinking of evidence via corroborative relationships enhances the scientific credibility of the findings (17).

Similarly, clinical trial data can establish connecting points with both classical literature and pre-clinical studies through reciprocal or interrelated corroboration. These connecting points—defined as overlapping content or shared intermediate facts—serve as bridges that integrate evidence from three distinct sources into a unified evidence chain (5). This chain not only improves the credibility and authenticity of each source but also helps establish the final fact to be proved.

## 3 Evolution and practice of the evidence chain paradigm in medicine

In contemporary medical research and clinical practice, there is an increasing demand for scientifically verifying medical facts and integrating diverse data sources to support optimal clinical decision-making. This has led to the progressive adoption of evidence chain theory, originally rooted in jurisprudence, within medical contexts (1). At its core, evidence chain theory provides a scientific framework for understanding and judgment by emphasizing corroboration theory, which highlights the interconnection among various forms of medical evidence. This review systematically explores the latest research developments and innovative applications of the evidence chain in medicine, illustrating how this interdisciplinary integration has helped standardize evidence evaluation and laid the groundwork for a more comprehensive and practical evidence-based practice model.

### 3.1 Establishment and development of evidence grading standards

**Figure 2:**
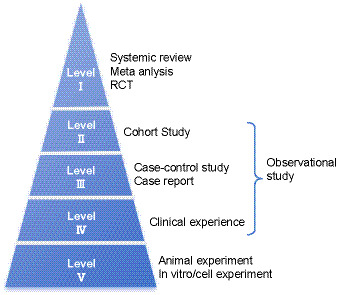
Evidence pyramid in evidence-based medicine.

Traditionally, medicine has relied heavily on individual clinical experience, mentorship from senior practitioners, and fragmented information from textbooks and medical journals. Consequently, some effective yet underrecognized therapies have been overlooked in clinical practice, while others—deemed theoretically beneficial but clinically ineffective or even harmful—have been widely adopted. EBM addresses these inconsistencies by classifying evidence into a five-level hierarchy based on reliability (18–20). At the top (Level I) are systematic reviews, meta-analyses, and randomized controlled trials (RCTs), while observational studies—such as cohort studies, case–control studies, case reports, and clinical experience—constitute Levels II to IV. Basic research, including cellular, animal, and *in vitro* studies, is categorized as Level V (Figure 2) (21). This pyramid-shaped structure is concise, visually intuitive, and widely disseminated. However, its rigid classification is overly mechanistic, often failing to accommodate the variability of individual clinical cases. Critically, it also lacks the capacity to adequately assess drug efficacy.

To address these limitations, the GRADE (Grading of Recommendations Assessment, Development and Evaluation) Working Group—comprising representatives from 19 countries and international organizations—introduced a unified framework for evidence grading and recommendation development in 2004 (22). The GRADE system provides a transparent, structured methodology with clear criteria for upgrading or downgrading evidence quality. It also introduced the GRADEpro software to assist users in evaluating evidence and generating recommendations (23). Central to GRADE is the concept of an evidence body, which includes all available evidence and evaluates its overall quality. Evidence is categorized into four levels (high, moderate, low, very low) based on the degree of confidence in the accuracy of efficacy estimates. Recommendations are classified as strong or weak, depending on the likelihood that following them will yield more benefits than harms (Table 1) (22, 24, 25).

**Table 1:** GRADE criteria for evidence quality and of recommendation strength.

	Grade	Describe	Study style
Evidence grade	High certainty	The true effect value approximates the estimated effect value.	RCTs; Observational studies with a two-level upgrade in quality
	Moderate certainty	The true value may be close to the estimate, but there remains a possibility of substantial difference.	RCTs with a one-level downgrade in quality; Observational studies with a one-level upgrade in quality
	Low certainty	The true value could differ substantially from the estimate.	RCTs with a two-level downgrade in quality; Observational studies
	Very low certainty	The true value is likely to differ considerably from the estimate.	RCTs with a three-level downgrade in quality; Observational studies with a one-level upgrade in quality; Series of case observations/case report
Recommendation grade	Strong recommendation	It is clearly demonstrated that the benefits of the intervention outweigh the harms or vice versa.	
	Weak recommendation	The benefits and harms are uncertain, or evidence of varying quality indicates that the benefits and harms are balanced.	

GRADE’s clear, actionable recommendations serve as more direct decision-making tools than traditional evidence levels, offering improved guidance for clinicians and researchers (26). It is currently the most widely accepted and effective system for evaluating medical evidence (27). Nonetheless, GRADE primarily focuses on clinical efficacy and does not accommodate the full spectrum of biomedical evidence. Furthermore, its criteria for modifying evidence quality can produce inconsistencies due to subjective researcher judgments.

### 3.2 Construction and application of integrated evidence chain

**Figure 3:**
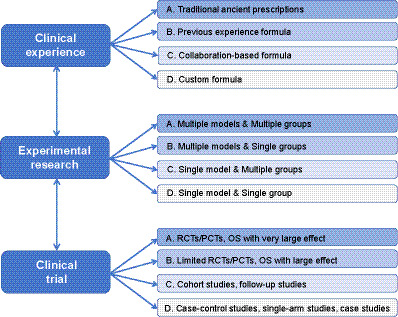
Diagram of integrated evidence chain.

Within the current EBM framework, it is particularly challenging for Traditional Chinese Medicine (TCM) to achieve high-quality evidence status, such as that represented by RCTs. Many time-honored TCM therapies cannot fully demonstrate their efficacy through conventional EBM assessments (28–30). For instance, Huoxiang Zhengqi *Liquid*, a classical formula for treating summer heat, has faced limitations in standard evaluations, which often overlook TCM’s emphasis on holistic bodily adjustment (31). Historically, little distinction was made between TCM and Western medicine in medical assessments, leading to the neglect of TCM’s core therapeutic principles. To overcome these barriers, Xiao et al. proposed the *Integrated Evidence Chain* (IEC) model (32). Grounded in clinical experience, the IEC systematically combines experimental research and clinical trials to construct a robust, evidence-based framework for efficacy evaluation. This approach not only provides scientific validation for classical prescriptions—enhancing their evidence level—but also facilitates TCM’s transition from empirical tradition to modern EBM. As a result, it drives the modernization and internationalization of TCM. The IEC emphasizes corroboration between clinical experience, experimental data, and clinical trial results, borrowing from jurisprudential theories of evidence relevance to construct a more comprehensive and interconnected evidence system (Figure 3) (33).

To establish the scientific validity of the IEC framework, Wei et al. conducted a detailed comparative analysis of the IEC and GRADE evaluation systems (34). They concluded that the IEC more effectively reflects the unique characteristics of TCM, including its diagnostic and therapeutic principles, material medical basis, R&D paradigms, therapeutic cognition, and evaluation modes. IEC has successfully integrated clinical experience with experimental research, forming a more holistic evidence evaluation system. Using the IEC approach, researchers have demonstrated that the combination of Xuefu Zhuyu *Decoction* with Western pharmacotherapy significantly improves outcomes in diabetes mellitus and its complications (35). The IEC method is now widely adopted in Chinese medicine research, where it is regarded as a scientifically grounded method of evidence generation. For instance, Wen et al. applied IEC to construct a comprehensive framework for “treating arthralgia syndrome from the spleen,” based on the theory that “spleen deficiency causes arthralgia syndrome” (36–38). This led to a scientific explanation of the effectiveness of the *Xin’an* Jianpi Tongbi prescription. In this context, IEC integrates multiple research pathways—including disease-syndrome correlation, clinical efficacy validation, multi-omics analysis, and mechanistic exploration—into a cohesive evidence chain. This paradigm supports the modernization of TCM by linking theoretical innovation to empirical validation, while also offering a roadmap for deeper integration of multi-omics and therapeutic target identification. In a separate effort, Gao et al. employed EBM methodologies to systematically evaluate the clinical efficacy and mechanistic basis of Guizhi Fuling Capsule across multiple diseases (39). By constructing an evidence chain centered around the concept of “blood stasis,” they not only identified the therapeutic advantages of the capsule for specific indications but also developed a novel decision-making framework for precise pharmacological use (40, 41). Their findings reaffirmed the modern applicability of core TCM principles such as “treating diseases based on syndromes” and “treating different diseases with the same method” (42). Furthermore, their cross-disease data emphasized the diagnostic and therapeutic significance of syndrome differentiation and pathogenesis in TCM. As a result, there is a growing consensus among researchers that IEC constitutes a scientifically robust methodology for evaluating drug and treatment efficacy in TCM (2, 43).

### 3.3 Construction and application of toxicological evidence chain

Our research group is addressing critical challenges in TCM toxicology, including insufficient analysis of complex toxicity mechanisms, unclear signal transduction pathways, and fragmented evidence in conventional toxicological studies. To overcome these limitations, we propose the Toxicological Evidence Chain (TEC)—a comprehensive framework specifically tailored for the study of toxic Chinese medicines (44–46). TEC anchors the source of toxic exposure, tracks observable toxicity signs, identifies mechanistic bases, pinpoints key inflection points in the toxicity cascade, and emphasizes the actual pathological damage to target organs. The methodology systematically reconstructs the progression of toxicity and supports robust causal inferences for toxic events.

**Figure 4:**
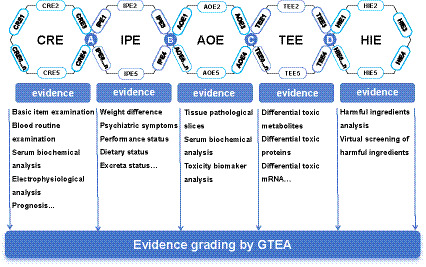
Diagram of toxicological evidence chain.

The TEC framework comprises five core components: 1. Clinical Risk Evidence (CRE): Derived from clinical data, including adverse event reports and online pharmacovigilance databases. 2. Injury Phenotype Evidence (IPE): Toxic phenotypes such as body weight changes, behavioral abnormalities, and toxicological status in model animals. 3. Adverse Outcomes Evidence (AOE): Substantial damage to target organs. 4. Toxic Events Evidence (TEE): Key signaling disruptions and metabolic alterations during toxicity progression. 5. Harmful Ingredients Evidence (HIE): Pharmacological components and substantiation associated with toxicological outcomes. These five bodies of evidence are interconnected through four key junctions, each aligned with a specific guiding question: A. Are CRE and IPE highly concordant—that is, do the clinical manifestations of toxicity and injury patterns in TCM closely mirror those observed at the animal level? B. Can IPE and AOE corroborate each other without logical contradiction? C. Are TEE and AOE causally or correlatively linked in the toxicological context? D. Based on TEE, can HIE be screened through correlation or linear correlation analysis? Each segment of the evidence chain is evaluated using the Grades of Toxicological Evidence Assessment (GTEA) system, which classifies evidence into hierarchical grades: S+/S−, A+/A−, B+/B−, and C+/C− (47). These components sequentially corroborate one another to construct a coherent and traceable chain of toxicological evidence (Figure 4).

Building on this framework, Kong et al. systematically applied the GTEA criteria to rate the evidence across each link in a TEC, constructing a nephrotoxicity evidence chain for podophyllotoxin (PPT) with clearly defined and quantifiable quality: IPE (B+), AOE (B+), TEE (C+/A), and HIE (B+) (47–50). Through integrated network toxicology analysis, they demonstrated that PPT modulates autophagy and oxidative stress via the PI3K/Akt/mTOR–Nrf2/HO1 pathway, leading to synergistic renal injury. Focusing on the previously undefined mechanisms underlying PPT-induced cardiotoxicity, Jiang et al. employed the TEC methodology in conjunction with multi-platform technologies to identify key regulatory nodes in the toxicity process (51, 52). Their results revealed that PPT disrupts energy metabolism and inflammatory homeostasis in cardiomyocytes via the SIRT1/PPAR/NF-κB signaling axis, ultimately contributing to cardiac damage. Furthermore, applying the TEC framework, researchers have explored the multi-organ toxicity mechanisms of PPT—including neurotoxicity, ovotoxicity, and enterotoxicity—offering standardized methodological references for analyzing complex toxicological profiles of other TCM and ethnic medicines (53, 54).

## 4 Discussion

### 4.1 Conclusion

In both medicine and jurisprudence, the structure and quality of evidence fundamentally reflect the underlying cognitive methodology. Whether dealing with factual relevance in legal cases or evidence stratification in EBM, the evidence chain holds a central methodological role (55). The clinician’s observations in EBM are akin to the “free evaluation of evidence” in legal reasoning. Cross-verification among multiple evidence levels and sources—such as basic research, clinical trials, and observational studies—is essential for assessing the validity and reliability of findings (56).

This paper explores the theoretical foundation of evidence chain theory, tracing its evolution within jurisprudence and clarifying core concepts such as the “connecting point” and the “corroborative evidence theory.” It further elucidates the theoretical rationale and internal logic underpinning the adaptation of this theory to the medical field. By systematically examining the development and application of evidence chains in medicine, the paper highlights their unique advantages in rapidly identifying key causal relationships, strengthening evidentiary support for factual conclusions, streamlining diagnostic processes, and optimizing clinical decision-making. In response to persistent challenges in evaluating the efficacy of TCM—notably the low level of evidence and the inadequacy of conventional evaluation systems to reflect TCM’s distinctive characteristics—the IEC model is proposed. IEC fully accounts for the diagnostic and therapeutic features of TCM as well as its subjective efficacy perception (28, 32). It integrates the experiential knowledge of TCM with the principles of EBM to construct a comprehensive evaluation model grounded in “clinical experience–experimental research–clinical trial.” This triadic framework addresses the limitations of over-reliance on RCTs and significantly enhances both the evidentiary strength and scientific recognition of classical prescriptions and other TCM practices rooted in clinical tradition. Consequently, IEC offers a reference framework for establishing an evidence-based registration and evaluation system tailored to TCM’s unique logic and clinical applications. Building on this foundation, the TEC extends the application of evidence chain theory to TCM toxicity research. It innovatively incorporates metabolomics, microbiomics, transcriptomics, and network toxicology to construct a five-dimensional systematic evidence model—“CRE–IPE–AOE–TEE–HIE.” This model enables multilevel analysis of the cascade toxicity pathways through which toxic TCM components affect various target organs. TEC thus presents a promising methodological breakthrough to address the longstanding issues of fragmented and inadequate evidence in TCM toxicity mechanisms.

As a core methodology for integrating multi-source data and reinforcing causal inference, evidence chain theory has emerged as a critical enabler of scientifically robust and practically effective clinical decision-making (57–59). Drawing on the clinical value of drug product positioning, Shen et al. pioneered the development of a medical literature evidence chain centered on this concept (60). They systematically integrated diverse evidence sources—including basic research, clinical studies, and quality control—thereby creating a framework that allows clinicians to more effectively communicate the true clinical utility of different medicines to target users. Their approach enhances diagnostic precision, improves alignment between physician and patient understanding, and boosts medication adherence. In a validation study by He et al., IEC demonstrated substantial clinical utility in diagnosing drug-induced liver injury (DILI) (61–63). By constructing a complete causal chain of evidence based on patient medication history, clinical symptoms, and biochemical test results, IEC overcame the limitations of traditional diagnostic methods. It significantly reduced false positives and missed diagnoses, thereby enhancing the sensitivity of DILI detection—particularly in the complex context of TCM-induced liver injury. Furthermore, the introduction of the electronic medical record (EMR) system has brought significant transformation to the construction and reinforcement of medical evidence chains. Through automated medical record systems, EMR enables the detailed integration and storage of multi-source clinical evidence, including patient demographics, clinical signs, MRI indicators, and laboratory results (64, 65). Notably, audit trail—as its core functionality—automatically records all operational details and modifications made by medical personnel within the system based on a precise timeline, thereby leaving clear and tamper-proof evidence at each process node. This mechanism not only provides a verifiable, transparent means to reconstruct the entire course of medical events, laying the foundation for high-quality medical evidence, but also effectively enhances the integrity, reliability, and traceability of the medical evidence chain (66, 67). In contrast, when the medical evidence chain cannot be fully established due to missing or broken critical links, clinical misjudgment and decision-making errors may occur. In a retrospective analysis of 367 patients with cervical spine injuries, Platzer et al. found that the fracture of imaging evidence was identified as the primary cause of missed or delayed diagnoses: among the 18 missed diagnoses, 28% resulted from incomplete imaging sequences, such as the absence of functional flexion/extension views, while 22% stemmed from poor image quality failing to reveal the injured segment. Ultimately, 8 patients (44%) developed neurological deficits due to missed diagnosis, including cases of complete quadriplegia (68). Additionally, Bunnell et al. reported a case of false-positive non-invasive prenatal testing (NIPT), where the pregnant woman’s NIPT results indicated the fetus had a high risk of 22q11.2 microdeletion. However, she declined invasive prenatal diagnostic procedures such as amniocentesis. The absence of this diagnostic verification step exposed the patient to prolonged psychological stress and potential risks associated with unnecessary pregnancy termination (69). Therefore, the ongoing exploration of methods and safeguards for establishing a comprehensive medical evidence chain is crucial for making correct clinical decisions, ensuring patient safety, and enhancing overall healthcare quality.

The core principles of evidence chain theory—relevance, completeness, and causal strength—are also reshaping clinical study design. Researchers increasingly favor multi-center, large-scale, prospective crossover trials to produce higher-level evidence, thereby constructing more robust, reliable, and persuasive evidence chains for clinical applications (70, 71). Ultimately, the deep integration of evidence chain theory into the medical domain not only enhances the precision of clinical diagnosis and treatment but also drives the evolution of clinical research from fragmented inquiry to systematic, theory-driven investigation.

### 4.2 Modern challenges

Although evidence chain theory has shown significant promise in the medical field, its practical implementation and in-depth application still face multiple challenges. Compared to its application in jurisprudence, the types of evidence in medicine—such as clinical symptoms, intervention outcomes, multi-omics data, and biomarkers—are more diverse and variable, and their generation mechanism, quality standards and evidence strength vary significantly. However, a standardized framework for evidence chain construction, including evaluation procedures, evidence classification, weighting schemes, and quality assessment criteria, remains underdeveloped. This lack of consensus can introduce considerable subjectivity into the construction and interpretation of medical evidence chains (34, 72, 73). Additionally, the core principles of TCM, which emphasize syndrome differentiation, rely on ambiguous, dynamic, and complex forms of evidence (e.g., symptom patterns and disease mechanisms). These characteristics are often incompatible with the linear causal logic that underpins most current evidence chain models (74, 75). Furthermore, the acquisition of high-quality evidence imposes stringent demands on routine laboratory screening systems. The high cost of advanced equipment significantly limits its widespread deployment, thereby hindering routine clinical application (76). What’s more, while clinical data recorded in the EMR system is highly detailed, interoperability barriers exist between EMR systems across different hospitals and even between departments within the same hospital. This readily creates data silos, leading to fragmented evidence information and inconsistent evidence quality. Consequently, it hinders the seamless construction and verification of medical evidence chains across institutions and regions (65). The audit trail of the EMR system, while ensuring operational traceability, presents significant technical challenges: how to effectively identify, parse, and transform the vast amounts of metadata it generates into reliable evidence that substantiates clinical decision-making logic, as well as how to guarantee the information security of the audit trail itself (77).

### 4.3 Future outlook

To address these challenges, there is an urgent need to integrate the distinctive features of medical evidence—particularly those unique to TCM—into the evidence chain framework. This includes establishing unified grading standards, developing quantitative assessment tools, and creating robust models for evidence evaluation. Collectively, these efforts will contribute to the development of a standardized, adaptable, and logically coherent evidence chain model suited to medical research. Looking ahead, advancements in artificial intelligence and big data analytics, along with the increasing availability of accessible technologies such as microfluidic chips and portable mass spectrometers, will facilitate the efficient acquisition of high-quality evidence (78). Simultaneously, leveraging shared platforms and cloud-based data analytics, efforts are focused on establishing standardized structures and interoperability frameworks for the EMR system. This will promote the normalization of data interfaces between healthcare institutions, offering a fundamental solution to the challenge of fragmented evidence. Additionally, advancing the deep integration of blockchain technology with EMR systems will help ensure the security of audit trails, providing underlying technical safeguards for the integrity and authenticity of audit logs. These measures collectively enhance the accessibility of high-quality evidence (79). Besides, the integration of surface plasmon resonance (SPR) technology with cellular and animal models offers multidimensional verification, providing more accurate molecular interaction data and significantly improving the authenticity and reliability of evidence materials (80). In the future, the application of evidence chain theory should place greater emphasis on dynamism and adaptability. It is essential to develop a “living evidence chain” system capable of real-time updates in response to disease progression and intervention outcomes. Such systems should be deeply embedded within the EMR platform and clinical decision support system, thereby achieving closed-loop management from evidence integration to clinical application and ultimately enhancing overall healthcare quality.

The interdisciplinary development of evidence chain theory is driving a paradigm shift in medical research—from an empirical approach to a closed-loop, evidence-based model. By incorporating jurisprudential logic to redefine standards for establishing medical facts, this approach offers a potential solution to the longstanding problem of fragmentation in medical practice. As such, it warrants broader scholarly attention to explore new paradigms for logically coherent, evidence-based clinical decision-making.
